# Charged Particle Therapy with Mini-Segmented Beams

**DOI:** 10.3389/fonc.2015.00269

**Published:** 2015-12-01

**Authors:** F. Avraham Dilmanian, John G. Eley, Adam Rusek, Sunil Krishnan

**Affiliations:** ^1^Department of Radiation Oncology, Health Sciences Center, Stony Brook University, Stony Brook, NY, USA; ^2^Department of Neurology, Health Sciences Center, Stony Brook University, Stony Brook, NY, USA; ^3^Department of Radiology, Health Sciences Center, Stony Brook University, Stony Brook, NY, USA; ^4^Department of Radiation Oncology, School of Medicine, University of Maryland, Baltimore, MD, USA; ^5^Brookhaven National Laboratory, Upton, NY, USA; ^6^NASA Space Radiation Laboratory, Upton, NY, USA; ^7^Department of Radiation Oncology, MD Anderson Cancer Center, Houston, TX, USA

**Keywords:** proton therapy, light-ion therapy, carbon therapy, proton minibeams, light-ion minibeams, carbon minibeams, tissue-sparing effect, interleaved carbon minibeams

## Abstract

One of the fundamental attributes of proton therapy and carbon ion therapy is the ability of these charged particles to spare tissue distal to the targeted tumor. This significantly reduces normal tissue toxicity and has the potential to translate to a wider therapeutic index. Although, in general, particle therapy also reduces dose to the proximal tissues, particularly in the vicinity of the target, dose to the skin and to other very superficial tissues tends to be higher than that of megavoltage x-rays. The methods presented here, namely, “interleaved carbon minibeams” and “radiosurgery with arrays of proton and light ion minibeams,” both utilize beams segmented into arrays of parallel “minibeams” of about 0.3 mm incident-beam size. These minibeam arrays spare tissues, as demonstrated by synchrotron x-ray experiments. An additional feature of particle minibeams is their gradual broadening due to multiple Coulomb scattering as they penetrate tissues. In the case of interleaved carbon minibeams, which do not broaden much, two arrays of planar carbon minibeams that remain parallel at target depth, are aimed at the target from 90° angles and made to “interleave” at the target to produce a solid radiation field within the target. As a result, the surrounding tissues are exposed only to individual carbon minibeam arrays and are therefore spared. The method was used in four-directional geometry at the NASA Space Radiation Laboratory to ablate a 6.5-mm target in a rabbit brain at a single exposure with 40 Gy physical absorbed dose. Contrast-enhanced magnetic resonance imaging and histology 6-month later showed very focal target necrosis with nearly no damage to the surrounding brain. As for minibeams of protons and light ions, for which the minibeam broadening is substantial, measurements at MD Anderson Cancer Center in Houston, TX, USA; and Monte Carlo simulations showed that the broadening minibeams will merge with their neighbors at a certain tissue depth to produce a solid beam to treat the target. The resulting sparing of proximal normal tissue allows radiosurgical ablative treatments with smaller impact on the skin and shallow tissues. This report describes these two methods and discusses their potential clinical applications.

## Introduction

The first utilization of segmented beams to increase superficial normal tissue tolerance to radiation was in grid therapy (1). The method was used in conjunction with 250-kVp orthovoltage x-rays in the early twentieth century to ameliorate the skin damage produced during radiation therapy of deeply seated tumors because of the low-dose penetration of the low-energy x-rays. The method involved positioning metal grids with openings as large as 2 cm on the patient’s skin. The resulting skin-sparing effect was solely due to the “dose–volume effect” according to which the tissue’s tolerance to radiation increases as its volume decreases (2, 3). Dose–volume is also the basis for all techniques of stereotactic radiosurgery [see, for example, Ref. (4)] in which high doses can be given to small targets, sometimes in a single-dose fraction.

**Figure 1:**
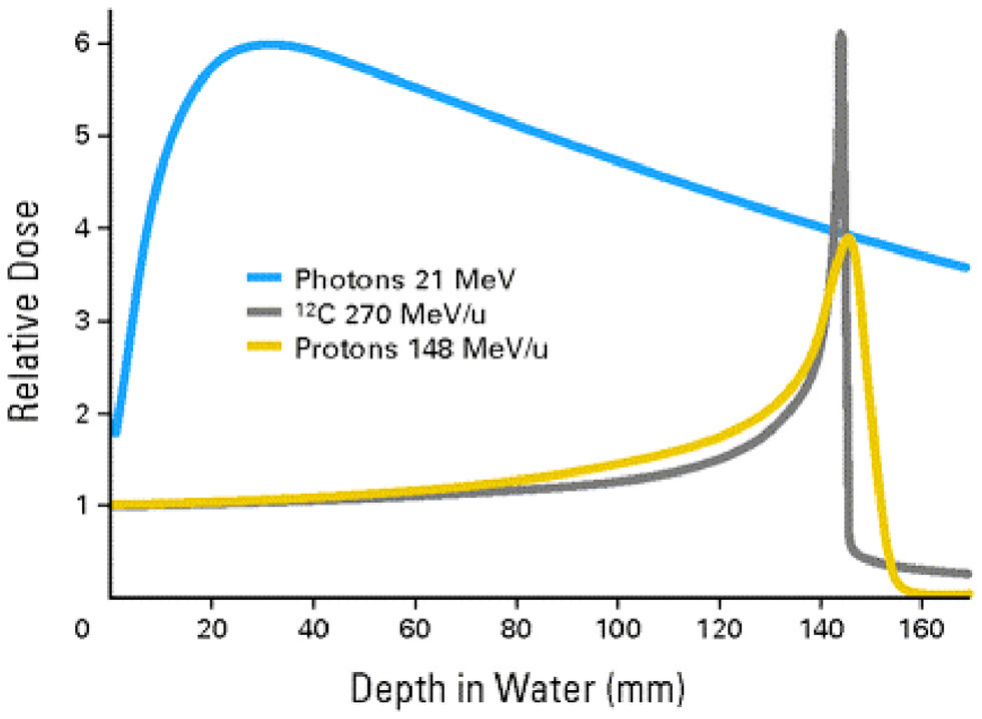
**Depth-dose distributions from 10 MV x-rays, proton beams, and carbon ion beams superimposed with each other for comparison**.

Although the introduction of the megavoltage (MV) x-ray machines into radiation therapy, which occurred in the middle of the twentieth century, solved the problem of damage to the skin and other proximal tissues from low-energy orthovoltage x-rays the challenge to find better beams for radiation therapy did not go away. This is because the dose distribution produced in tissues from MV x-rays is far from ideal. As seen in Figure 1, they give unnecessary dose to the normal tissues surrounding the target both proximal and distal to the target. Furthermore, their lateral dose penumbra is very large. Although proton and carbon ion beams clearly produce a better dose confinement at the target because of their Bragg-Peak feature, they both lack the shallow-tissue-sparing effect of high-energy x-rays, which is considered a significant shortcoming.

Two major developments that occurred since the time of grid therapy in experimental radiation therapy indicate a great potential for segmented beams at much smaller beam sizes than those used in grid therapy to improve radiation therapy. First, Zeman et al. (5–7), studying the tolerance of the mouse cerebellum to pencil beams of 25-μm to 1-mm diameter in the 1950s, showed that the mouse cerebellum tolerates the smaller beams considerably better than 1-mm beams. Specifically, microscopic beams of 25 and 75 μm did not cause tissue necrosis (i.e., loss of the tissue’s blood perfusion) at doses up to 10,000 Gy, although they lead to neuronal cell death in their direct beam path, while 1-mm beams of 120–300 Gy literally ablate brain tissues at certain time points within the 24–120 days post-irradiation; these results include 300 Gy (5) and 140 Gy (7) ablations at 24 days and 280 Gy ablation at 120 days (6). Second, it was shown in the 1990s that the rat cerebellum tolerates arrays of parallel, very thin planes of synchrotron x-rays at very high doses. Specifically, Slatkin et al. showed at the National Synchrotron Light Source (NSLS), Brookhaven National Laboratory (BNL) that arrays of parallel, 37-μm synchrotron x-rays, spaced 75 μm on-center (microbeams) of ~50 keV median energy were tolerated by the rat cerebellum at up to 250 Gy in-beam in-depth without producing any visible effect on the H&E-stained brain tissue 3 months later (8). The excitement produced by the observed tissue-sparing effect led to the start of a new line of research at both the NSLS and the European Synchrotron Radiation Facility (ESRF) in Grenoble, France, called microbeam radiation therapy (MRT) (9–16). The early experiments in both these labs included measuring the tolerance of the central nervous system (CNS) in very young animals to very high doses of x-ray microbeams; these included brains of duck embryos irradiated to 120 Gy (9) and cerebella of suckling rats and weanling piglets irradiated to 300 Gy (13). We note that it has become a convention in the field of MRT to call beams with 0.3-mm size and larger “minibeams.”

The effects observed in the above studies were categorically different from grid therapy in two ways. First, it showed that tissues much deeper that skin can tolerate huge doses, and second, as shown in the presentation of mechanistic bases for this larger tissue tolerance later in this report, the effect goes far beyond the dose–volume effect and involves what is called “prompt microscopic biological repair effect” including capillary blood vessel repair (10–12, 14, 16).

**Figure 2:**
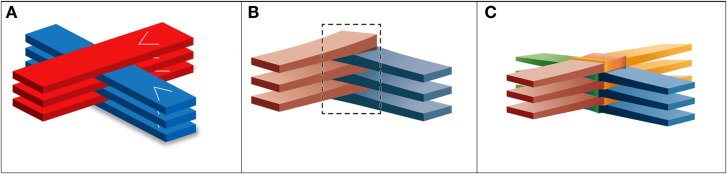
**Schematic views of interleaved x-ray minibeams and two- and four-directional interleaved carbon minibeams**.

The next major development in the field occurred some 10 years later when it was shown at the NSLS that arrays of synchrotron X-ray microbeams as thick as 0.68 mm (minibeams) still retain much of their tissue-sparing effect in the rat spinal cord and brain (17). Furthermore, it showed that two such arrays aimed at the target from 90° angles, with gaps between the beams equal to the minibeams’ thickness, can be “interleaved” (or “interlaced”) to produce a solid radiation field at the target (17) (Figure 2A). The method was used with 0.68 mm beams to ablate a 3-mm target in a rat brain at 120 Gy with a solid interlaced beam at the target; very little damage was observed in the H&E-stained tissue outside the target (17).

**Figure 3:**
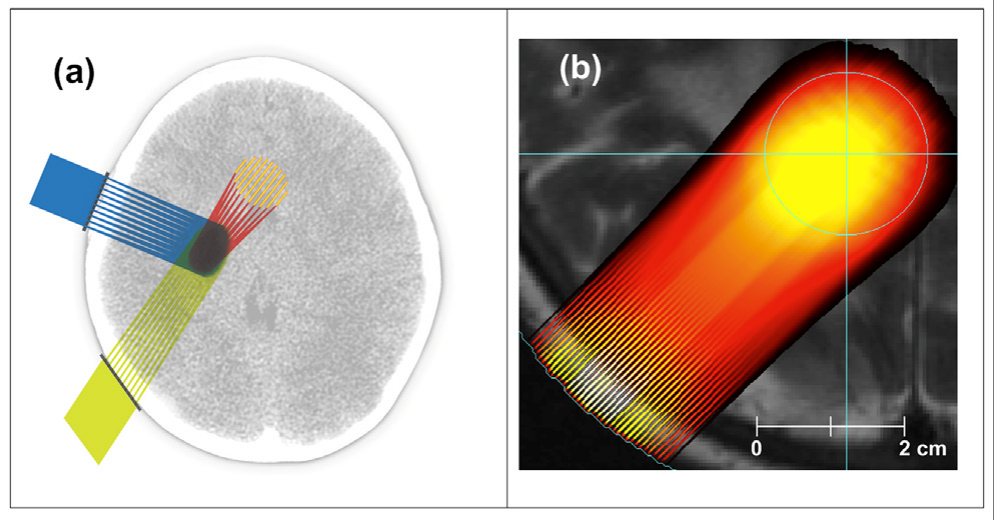
**Treating a target in the brain with proton minibeams: (A) schematic view of exposures from three orthogonal directions, (B) Monte Carlo simulation of a single exposure on the background of a brain MRI**.

The above finding about the sizable tissue-sparing effect of minibeams as thick as 0.7-mm opened the way for charged particle minibeams to be evaluated in similar preclinical studies. The first such evaluation was with carbon ion minibeams at the NASA Space Radiation Laboratory (NSRL) at BNL. They were used in the “interlaced” (or “interleaved”) geometry (Figures 2B,C) to ablate a small target in the rabbit brain (18). The rabbits evaluated in 6 months with contrast-enhanced magnetic resonance imaging (MRI) and histology showed virtually no damage to the surrounding tissues. The method, however, could not be implemented with protons and light ions because of their excessive broadening with tissue depth. However, it was shown through dosimetric measurements with proton minibeams and Monte Carlo simulations with proton and light-ion minibeams that minibeams in such arrays can be designed to merge with their neighbors at a certain depth in the subject to produce a solid beam for treating targets while sparing the skin and other shallow tissues (19) (Figure 3).

## Materials and Methods

Although the minibeams’ decline of tissue sparing with increasing beam size is gradual; nevertheless, any treatment planning with minibeams will require defining an upper size limit for the minibeams’ usage. For the following reasons, we suggest that this limit will be set at 0.7 mm. First, studies with 0.68 mm planar synchrotron x-ray minibeams with 0.68 mm gaps between them showed that irradiation of nearly the entire rat brain with these beam arrays at 170 Gy beams were greatly tolerated for the 7-month period of observation (17). Specifically, not only did the rats not demonstrate any neurological or histological deficits but they also gained weight normally (17). On the other hand, studies by Zeman et al. with 1 mm diameter 25-MeV deuteron pencil beams demonstrated complete tissue ablation of the mouse cerebellum at doses as little as 140 Gy within 280 days. These results indicate that the minibeams’ tissue-sparing effect declines quite sharply at beam dimensions beyond 0.68 mm. Although 0.7-mm might be considered a conservative upper limit, its choice is justified because possible clinical use of charged particle minibeams will require very high incident doses to produce adequate target doses.

The incident minibeam width of 0.3-mm used in all experimental and simulation studies presented below was chosen in the context of the above consideration for the upper limit of the allowable minibeam size. It provides us with some dynamic maneuvering range of the beam thickness before reaching the upper limit. We also point out that choosing incident beams smaller than 0.3 mm is technically difficult, and at the same time it does not add much to that maneuvering range. As for the choice of the array’s minibeam spacing, an on-center value of 0.7-mm seems to ideal for most cases because it makes the minibeams merge with each other immediately after they reach their maximum allowable size.

### Interleaved Carbon Minibeams

The rabbit study described below was carried out in accordance with the recommendations of the Institutional Animal Care and Use Committee (IACUC) of BNL. BNL is accredited by the American Association for Accreditation of Laboratory Animal Care, Inc. (AAALAC). The protocol was approved by the BNL’s IACUC.

**Figure 4:**
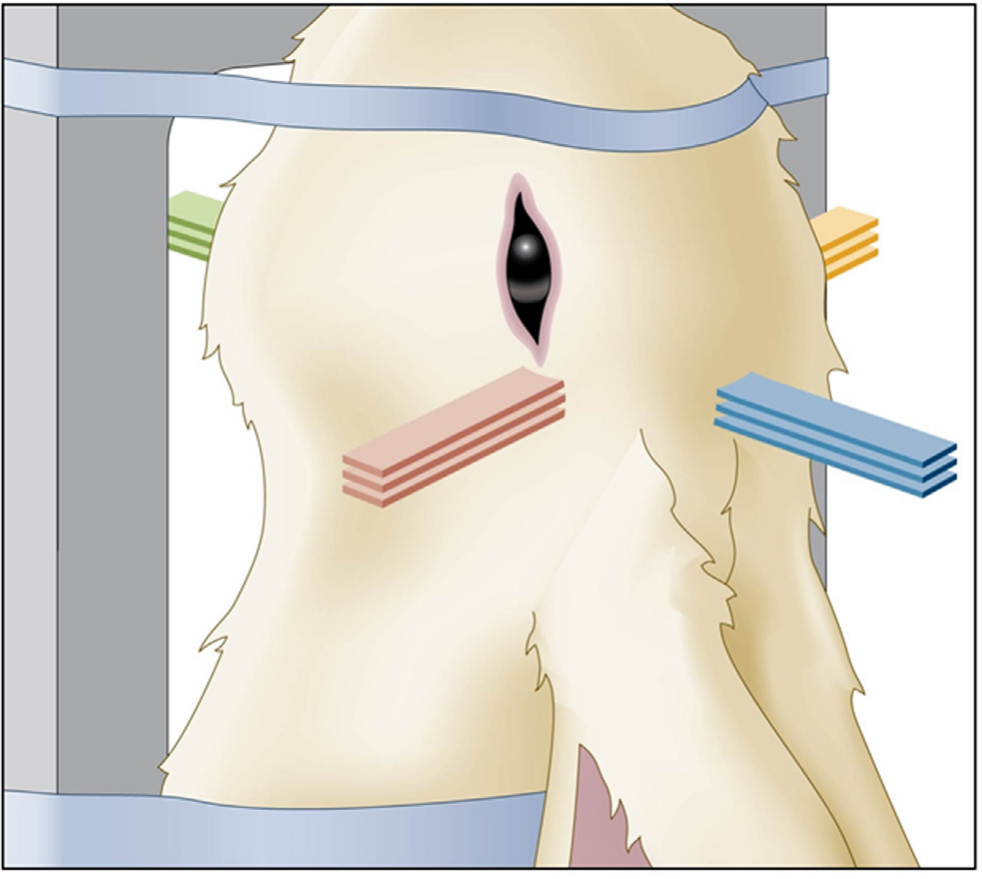
**Schematic view of the rabbit head irradiated with four-directional interleaved carbon minibeams**.

**Figure 5:**
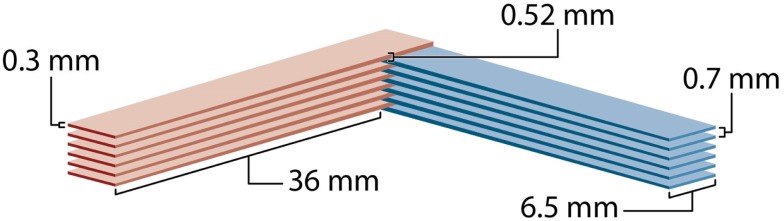
**Two-directional interleaved carbon minibeams drawn to scale for the rabbit experiment**.

Figure 4 shows the schematic view of the four-directional interleaved carbon ion minibeams used to ablate a 6.5-mm target in a rabbit brain (18). The details were the following. The minibeams’ incident-beam thickness was 0.3 mm, and their beam spacing on-center was 1.05 mm on-center; this spacing was much larger than twice the incident-beam thickness to accommodate the gradual minibeam broadening in tissues. Figure 5 shows a “to-scale” presentation of two of the four carbon minibeam arrays used in the study. The study used 124–135 MeV/nucleon carbon energies to create the spread-out-Bragg-peak (SOBP). The total dose produced at the target from all four directions in the SOBP was 40 Gy physical absorbed dose, which corresponds to 120 photon-equivalent Gy (GyE) using an average relative biological effectiveness value of 3.0 at the target. The following dosimetry account shows that this dose was produced by minibeams of 14 Gy in-beam incident physical dose in each of the four interleaving arrays. First, it was shown (18) that the particular geometry involving 14 Gy “pedestal” incident dose leads to 20 Gy physical dose at the SOBP. Therefore, the target dose would have been 20 Gy (and not 40 Gy) if we had only two interleaving arrays (see Figure 2B for geometry). The 40-Gy physical target dose was produced by virtue of having a four-directional incident-beam geometry (Figure 2C).

### Proton and Light-Ion Minibeams

Figure 3A is a schematic representation of treating a brain tumor with three minibeams arrays aimed at the target from mutually orthogonal angles, whereas Figure 3B presents the results of Monte Carlo simulations of minibeams’ merging in one such array and overlaid on a clinical MRI scan of the brain. The method’s physical feasibility was established by measurements at MD Anderson Cancer Center and by Monte Carlo simulations (19).

The measurements at the MD Anderson Proton Therapy Center included the broadening rate on chromographic film of a 109-MeV proton pencil beams with 0.3-mm incident-beam diameter. The beams were produced by a pinhole collimator made of a 1-cm thick tungsten–copper alloy and the measurements were carried out using a stack of radiochromic films interspersed with 2-mm plastic sheets positioned downstream of the collimator. The film stack was irradiated to 10 Gy peak dose to measure the minibeam’s broadening (19). In another measurement, a pattern of 100 MeV planar proton minibeams of 0.3-mm thickness, spaced 1-mm on-center was captured on a chromographic film (19); the minibeam array was produced using a 5-cm thick tungsten multislit collimator.

## Results

### Interleaved Carbon Minibeams

The carbon minibeams’ rate of beam broadening with tissue depth is quite suitable for interleaved carbon minibeams of target sizes such as that of the brain but not for larger sizes. In this regard, the criterion is that the minibeams’ width at the proximal side of the target should not substantially exceed 0.7 mm where their tissue-sparing effect starts to gradually diminish. For example, in the above study, a minibeam width of 0.525 mm was produced at the target’s proximal side, which was 36-mm deep (tissue equivalent) (18). This broadening is in agreement with the criterion that in the method’s future clinical application the maximum allowable depth of the proximal side of the target is 6.5 cm; heavier ion beams such as oxygen might be used for treating deeper tumors (18).

The rabbit, observed for 6 months, did not show any behavioral deficits. Contrast-enhanced MRI showed extensive gadolinium contrast leakage only from the target tissue but not the surrounding tissues. Furthermore, H&E tissue staining showed a necrotic target tissue with no sign of damage and a slide only 5.5 mm away from the center of the target showed no damage along the four interleaving beam arrays (18).

### Proton and Light-Ion Minibeams

**Figure 6:**
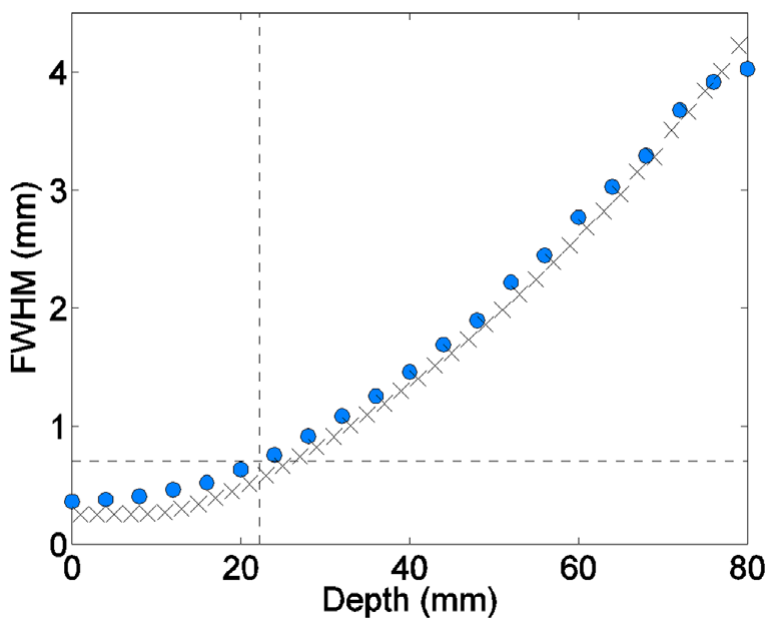
**Proton pencil minibeams’ broadening for 109 MeV beam energy (circles: film measurements; x marks: the simulations in water)**.

Figure 6 shows the measured minibeam broadening rate for 109-MeV proton pencil beams superimposed with the Monte Carlo simulations of the same minibeam width in water. The experimental minibeam width was defined as the full width at half-maximum of the beam’s image on the digitized film. The figure shows that the measured and the simulated 0.7-mm beam width for this beam energy are reached at 22 and 23.5 mm, respectively (19).

**Figure 7:**
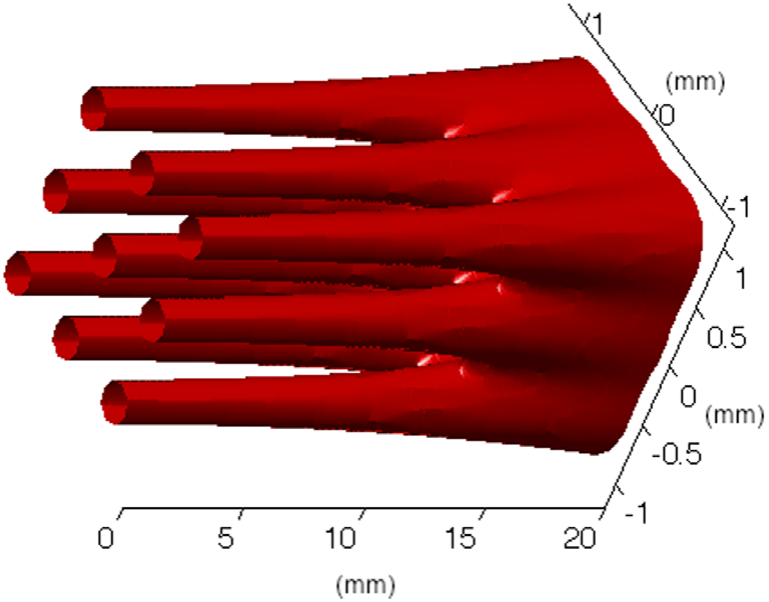
**Monte Carlo simulations of a three-dimensional pattern of proton pencil minibeams converging at a target**.

**Figure 8:**
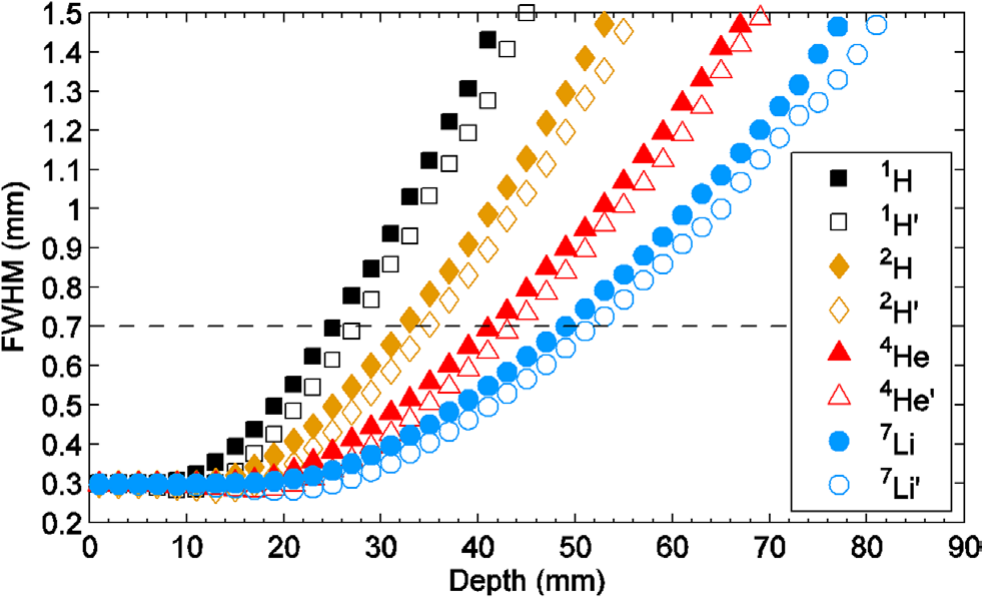
**Monte Carlo simulation of the broadening rates in water of 0.3-mm pencil-shaped (solid marks) and planar (hollow marks) minibeams of protons, deuterons, and He-4 and Li-7 ions, all at the incident-beam energy, leading to 10-cm depth in water**.

Figure 7 shows a schematic view of a three-dimensional minibeam converging in an array of proton pencil minibeams with 0.3-mm incident-beam thickness and 0.7-mm beam spacing on-center. It demonstrates the physical feature of the event, emphasizing the effect of the acceleration of the broadening rate as the beam approaches the merging point. This acceleration can be clearly seen also in Figure 8 that shows the Monte Carlo simulation results of the broadening rates of 0.3-mm pencil-shaped and planar minibeams of protons, deuterons, and helium 4 and lithium 7 ions penetrating water at incident energies leading to 10 cm water depth. The acceleration effect of the beam broadening is clearly manifested by the curving-up feature of the curves.

**Figure 9:**
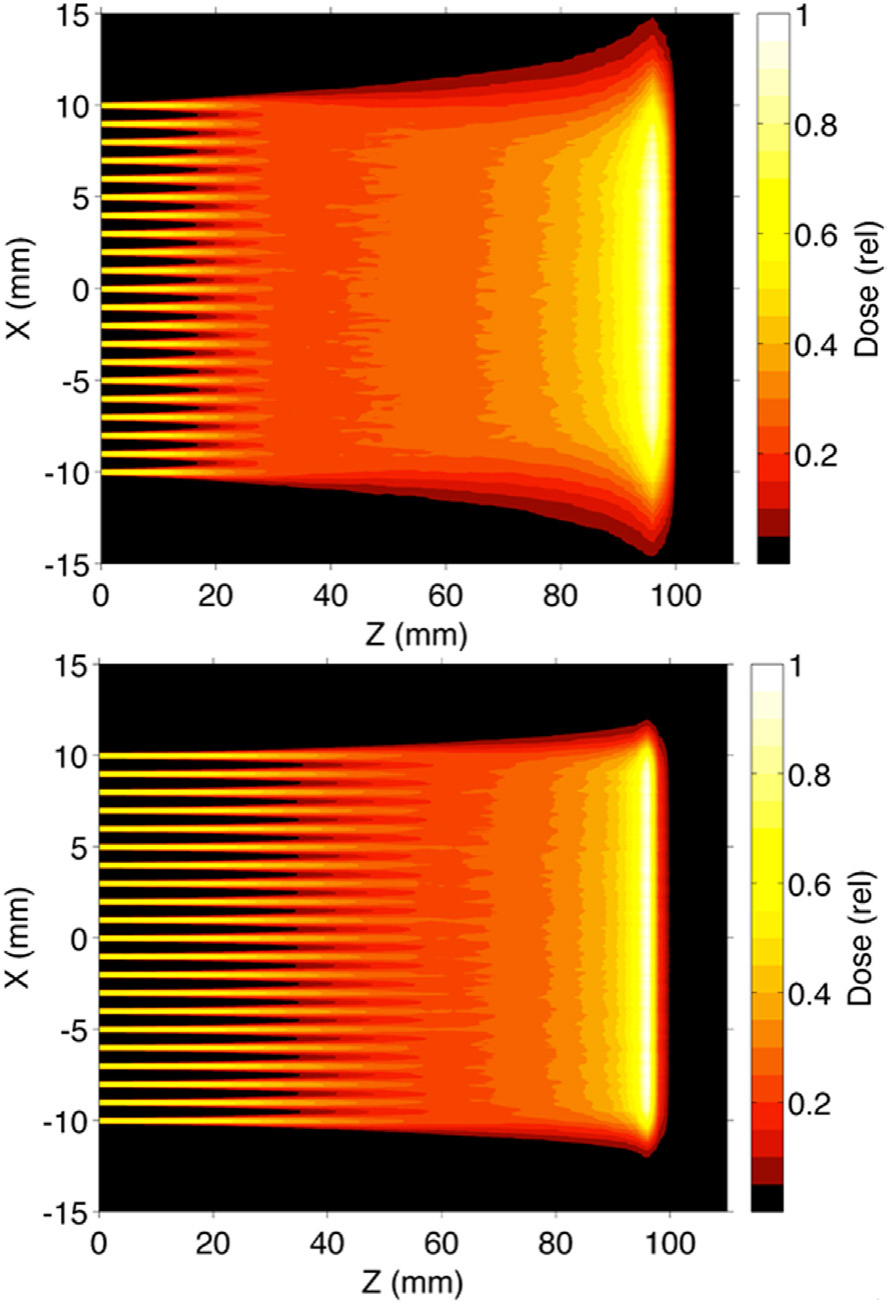
**Monte Carlo simulation of the dose distribution with depth in water of the 0.3-mm proton and Li-7 planar minibeam arrays of Figure 8, spaced 1.0-mm on-center**.

Finally, Figure 9 shows the two-dimensional pattern of the minibeam broadening for the planar minibeams of protons and lithium 7 of Figure 8, with 1.0 mm minibeam spacing on-center. The simulations indicate that water depths at which the minibeams in these two arrays fully merge with their neighbors are 29 and 56 mm, respectively (19).

## Discussion

The data and perspectives presented in this section are mostly discussions related to the clinical potential of interleaved carbon minibeams and gradually broadening arrays of proton and light-ion minibeams that converge at the tumor. In the course of this discussion, comparison is made with the radiation therapy methods currently in clinical use, including MV x-rays, protons, and carbon ions. In this context, an important subject is the incident dose in the individual minibeams, which are clearly much higher than those used in the incident conventional beams to make up for the non-irradiated tissue slices residing between the incident minibeams.

Comparing interleaved carbon minibeams with conventional carbon therapy the advantage is smaller radiation impact on the non-targeted tissues, particularly the proximal tissues. This can allow reducing the number of dose fractions and/or increasing the target dose, which can be important in treating radioresistant tumors.

Comparing proton minibeams with conventional proton therapy, the advantage is mostly saving the skin and the proximal tissues. The method’s application can include the facilitation of dose hypofractionation. But, most importantly, the method can reduce the damage to certain eloquent and/or radiosensitive organs in the brain such as the cerebral cortex that is involved in gliogenesis, especially in children (20), and therefore its sparing may reduce cognitive deficits. The method can also reduce the integral biological brain dose, another important factor in reducing cognitive deficits when targets in the brain are treated (21–23).

Comparing both methods with conventional MV x-ray methods such as intensity-modulated radiation therapy (IMRT), radiosurgery, and stereotactic body radiotherapy (SBRT), the advantages include all advantages of the corresponding conventional charged-particle methods together with the above advantage of the minibeam-implemented charged-particle methods. For both methods that include the Bragg-peak feature of the particle methods, while for carbon therapy, it also includes larger target RBE and smaller lateral dose falloff.

### Mechanisms Underlying the Tissue-Sparing Effect of the X-Ray Microbeams and Minibeams

The microbeams’ tissue-sparing effect is thought to be based on two phenomena, namely, the “dose–volume effect” (2, 3) and the “prompt, microscopic, biological repair effect” (10–12, 14, 16). As indicated above, dose–volume effect means that the tissue’s tolerance to radiation increases as the volume of the irradiated tissue decreases (2, 3). The effect, valid for any target size, has been the basis for grid therapy (1) and stereotactic radiosurgery (4). On the other hand, the prompt, microscopic, biological repair is specific to microbeams and minibeams at beam sizes below about 0.7 mm. It is primarily related to the fast (within hours and days) repair of the capillary blood vessels via the regeneration of angiogenic cells surviving between the microbeams and minibeams (10, 12).

### Quantitative Estimation of the Magnitude of the Minibeams’ Tissue-Sparing Effect

One can quantitatively compare the maximum tolerance of the rat brain to minibeams and solid beams using Ref. (17, 24), respectively. While in the former, the entire brain tolerated, both behaviorally and histologically, 0.68 mm planar minibeams with 0.68-mm gaps between them at 170 Gy for the 7-month observation period (17), the latter’s local 22.5-Gy solid beam irradiation led to “histological evidence for the development of necrosis in the white matter after a latent period of >26 weeks” (24). This puts the dose-tolerance advantage of minibeams to that of solid beams over 7.5-fold.

### Magnitude of the Incident-Beam Doses in Clinical Interleaved Carbon Minibeams

Comparing the incident dose in 0.3-mm carbon minibeams spaced 0.7-mm on-center to carbon solid beams, both delivering the same target dose from two 90° incident directions, the minibeams’ incident dose should be 4- to 4.7-times larger. This is because (a) doses from 90°-pairs of interleaving arrays do not add up and (b) minibeams’ dose is diluted by 2- to 2.33-fold (0.7/0.3 ratio for 0.3 minibeams broadening to 0.7 mm just before the target) on the way to the target because of their beam broadening. This high dose of 0.3-mm carbon minibeams should be well tolerated by the skin and the other proximal tissues because as discussed above the tolerance advantage of 0.68-mm minibeams with 0.68-mm gaps between them over solid beams in about 7.5:1.0, and therefore that of 0.3 minibeam with 0.4-mm gaps between them should be even higher, probably over 10-fold. Therefore, a 4.7-fold higher dose in the incident minibeams compared to a solid beam should be tolerable.

### Magnitude of the Incident-Beam Doses of Protons and Light-Ion Minibeams

Comparing the incident dose in 0.3-mm proton or light-ion minibeams spaced 0.7-mm on-center to that of proton or light-ion solid beams, both delivering the same target dose, the incident minibeam dose should be 2.3-fold higher when using planar minibeams and 6.9-fold higher when using pencil-shaped minibeams. This is because of the much smaller yield of the collimator producing pencil-shaped beams. Although using the above argument for the much higher tissue tolerance of the tissues to minibeams than solid beams the proton minibeams should still be tolerated, the clinical utilization of the pencil-shaped beams seems less attractive. This problem, however, is solved with the use of light-ion minibeams such as He-4 or Li-7 because the smaller rate of minibeams broadening allows the use of smaller than 0.7-mm beam spacing on-center to still spare several centimeters of proximal tissues. In that regards, the collimator yield for 0.3-mm beams spaced 0.5-mm on-center is 28.3% compared to 14.4% for 0.7-mm pencil-beam spacing, and the minibeams dose will be only be 3.5-fold higher than that from incident solid beams.

### Comparing the Dose–Depth Distributions in a Brain Tumor Phantom Produced by MV X-Rays, Solid Proton Beams, and Proton Minibeams

**Figure 10:**
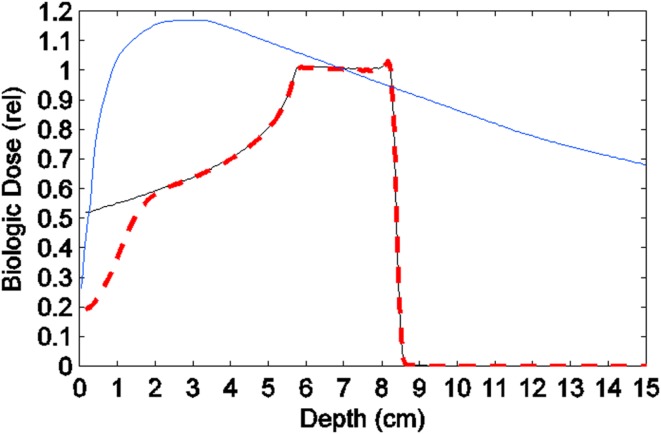
**Comparing the depth-dose distributions in a phantom of (a) a 10-MV x-ray beam, (b) an incident solid proton beam with its spread-out Bragg-peak, and (c) estimated biological dose from an incident proton minibeam array undergoing the same pattern of Bragg-peak spreading as above**.

Although the “biological dose” from incident proton minibeams cannot be calculated without detailed experiments, a rough estimate of the dose can be made through our knowledge of the magnitude of minibeam’s tissue-sparing effect. Here, we compare the dose–depth distributions in a 15-cm deep water phantom of (a) a 10-MV x-ray beam, (b) an incident solid proton beam with its SOBP over a 2.5-cm target starting at 5.5-cm depth from the surface, and (c) estimated biological dose from an incident array of proton minibeams merging into a solid beam at 2.5-cm depth, which has undergone the same pattern of Bragg-peak spreading as the solid proton beam (Figure 10). As seen in this figure, the entrance biological dose of the proton minibeams is set to 1/3 of the physical dose of the solid proton beams. This estimate is the product of two factors. First, the incident dose in each minibeams should be 2.3 times (for 0.3-mm minibeams spaced 0.7-mm on-center) that of the solid beam to make up for the un-irradiated slices of tissue between the minibeams. Second, the magnitude of the tissue-sparing effect for 0.3-mm minibeams is estimated to be sevenfold from the earlier discussion in this section. This makes the biological dose compared to the solid beam of protons 2.3/7.0 = 0.33. In other words, despite the 2.3-fold larger in-beam physical dose needed, still the biological dose is only 33% of the physical dose of the incident solid proton beam. The figure pictorially demonstrates the tissue-sparing effect of the proton minibeams in shallow tissues and their contribution to reduce the integral brain dose.

### Potential Clinical Challenges

The use of interleaved carbon minibeams requires the immobilization of both the proximal tissues and the target tissues. This limits the method’s clinical applications to benign and malignant tumors of the brain and spinal cord, neurological targets, head-and-neck tumors, breast cancer (with the breast immobilized), and tumors of the spinal column and the extremities. The neurological targets may include those that give rise to epilepsy, trigeminal neuralgia, tremor, and obsessive compulsive disorder. As a remote possibility, the method might be applicable to treat tumors of the chest and abdomen such as those of the liver and pancreas when administered under anesthesia as a single fraction.

However, the requirements for proton and light-ion minibeams are much more relaxed. There, the immobilization requirements apply only to the proximal tissues that have to be spared, and not to any deeper normal tissues or the target. Since the tissue-sparing range of proton minibeams is only 2–3 cm tissue depth, abdominal target can be treated by immobilizing the shallow tissues by physical means using the multislit collimator or a frame positioned in front of it. Of course, all possible applications indicated above for carbon minibeams can also be treated with proton and light-ion minibeams.

A legitimate concern regarding proton and light-ion minibeams pertains to the dose to the patient from the neutrons produced in the multislit collimator. Best estimates of this dose are that they are a very small fraction of the incident dose, even when accounting for biological effectiveness of neutrons. In addition, they can be further reduced by introducing an air gap between the collimator and the subject’s skin. This issue was further discussed more recently with the journal following a Letter to the Editor (25). The conclusion was that for protons and for a 5-cm gap between the collimator and the skin the dose will be about 1% of the peak target dose. Because the gap can be made larger that 5-cm we do not expect this issue to be a major factor in charged particle minibeam therapy.

### Ease of Clinical Implementation

Both methods can be readily integrated into current clinical practice of carbon therapy, proton therapy, or light-ion therapy by positioning a multislit collimator in the path of the broad incident beam. They are entirely compatible with passively scattered beam or spot-scanned beams. Interleaved carbon minibeams require two of four 90° irradiations, while the treatment with protons and light-ion minibeams could also ideally be done at 90° angles to avoid production of tissue irradiations with shallow-angle minibeam exposures producing incomplete tissue-sparing in these areas, called minibeam star artifacts. Also, two fixed horizontal beam-lines aiming at the target from 90° could be used for simultaneous administration of two arrays. Finally, both methods can be administered in the raster scanning mode, thus further reducing the dose to the proximal tissues.

## Author Contributions

FD participated in all studies and in writing the manuscript. JE participated in the proton minibeam studies and in writing the manuscript. AR participated in the interleaved carbon minibeam studies. SK participated in the proton minibeam studies an in writing the manuscript.

## Conflict of Interest Statement

F. Avraham Dilmanian has a patent on interleaved carbon minibeams. F. Avraham Dilmanian, John G. Eley, and Sunil Krishnan have a pending patent on minibeams of protons and light ions. Adam Rusek declares that the research was conducted in the absence of any commercial or financial relationships that could be construed as a potential conflict of interest.
